# Haloarchaea and the Formation of Gas Vesicles

**DOI:** 10.3390/life5010385

**Published:** 2015-02-02

**Authors:** Felicitas Pfeifer

**Affiliations:** Microbiology and Archaea, Department of Biology, Technische Universität Darmstadt, Schnittspahnstrasse 10, 64287 Darmstadt, Germany; E-Mail: pfeifer@bio.tu-darmstadt.de; Tel.: +49-6151-1623670; Fax: +49-6151-1623672

**Keywords:** halophilic Archaea, gas vesicle formation, gene regulation

## Abstract

Halophilic Archaea (Haloarchaea) thrive in salterns containing sodium chloride concentrations up to saturation. Many Haloarchaea possess genes encoding gas vesicles, but only a few species, such as *Halobacterium salinarum* and* Haloferax mediterranei*, produce these gas-filled, proteinaceous nanocompartments. Gas vesicles increase the buoyancy of cells and enable them to migrate vertically in the water body to regions with optimal conditions. Their synthesis depends on environmental factors, such as light, oxygen supply, temperature and salt concentration. Fourteen gas vesicle protein (*gvp*) genes are involved in their formation, and regulation of *gvp* gene expression occurs at the level of transcription, including the two regulatory proteins, GvpD and GvpE, but also at the level of translation. The gas vesicle wall is solely formed of proteins with the two major components, GvpA and GvpC, and seven additional accessory proteins are also involved. Except for GvpI and GvpH, all of these are required to form the gas permeable wall. The applications of gas vesicles include their use as an antigen presenter for viral or pathogen proteins, but also as a stable ultrasonic reporter for biomedical purposes.

## 1. Introduction

The moderately to extremely halophilic Archaea (Haloarchaea) are adapted to salty environments with sodium chloride concentrations ranging from 1 M up to saturation at 5.3 M. They are heterotrophic, often facultatively anaerobic microorganisms living on amino acids. Bioinformatic analyses of 10 haloarchaeal and more than 1000 bacterial reference genomes imply that a common haloarchaeal ancestor, the “founder” haloarchaeon, evolved from an anaerobic methanogen by acquiring 1047 bacterial genes, thus expanding the physiological properties to an aerobic organotroph [1]. The transferred genes mainly derive from Actinobacteria and are responsible for catabolic metabolism, membrane transporters and components required for oxygen respiration [1,2]. Lateral gene transfer is still a special feature of the Haloarchaea, but their aerobic lifestyle appears to be a consequence of a single mass acquisition of bacterial genes.

**Figure 1:**
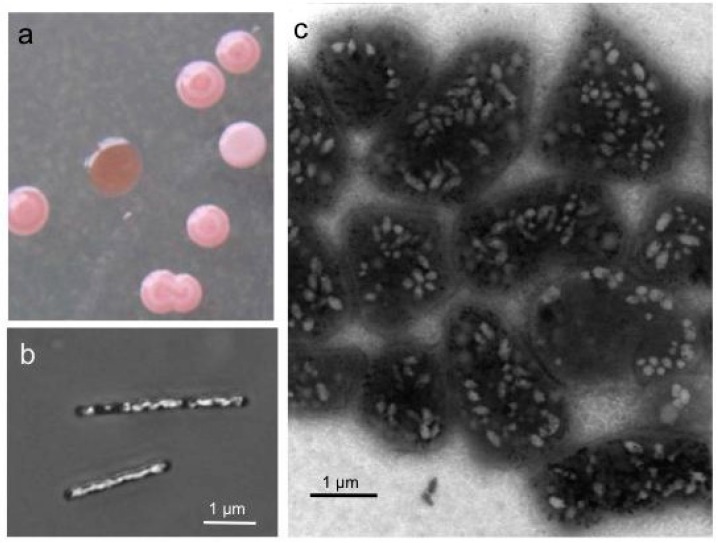
Colonies (a) and cells of *Halobacterium* (*Hbt.*)* salinarum* producing gas vesicles (b,c). (**a**) Colonies on solid media grown for one week at 40 °C and three weeks at room temperature. Vesicle (Vac^+^) cells form pink white colonies, whereas colonies of Vac^− ^mutants are red and transparent. (**b**) Cells grown in liquid media observed by phase-contrast light microscopy. (**c**) Cells of a Vac^+^ colony investigated by transmission electron microscopy. The pleomorphic shape of the cells grown for three months on solid media differs from the rod-shaped cells seen in liquid culture.

The first haloarchaeon characterized in the laboratory was *Halobacterium* (*Hbt.*)* salinarum* (formerly *Hbt. halobium*), isolated as a contaminant from salted fish in 1919 [3]. All other Haloarchaea derive from salt lakes, salt flats or solar salterns. *Hbt. salinarum* uses light-driven ion pumps (proton pump: bacteriorhodopsin; chloride pump: halorhodopsin; plus sensory rhodopsins) as special energy conversion and sensing systems, and similar light-driven proton pumps are also present in marine proteobacteria [4,5]. Bacteriorhodopsin is produced under microaerobic conditions and forms almost crystalline purple patches in the cytoplasmic membrane of *Hbt. salinarum* (purple membrane, Pum)*.* Haloarchaea also contain C50 carotenoids (bacterioruberins, Rub) leading to red colonies on agar plates. The possession of gas vesicles (Vac) turns the colony color into pink and opaque, as opposed to the red transparent colonies of Vac^−^ mutants (Figure 1a). Blooms and biofilms of Haloarchaea color brines and salt lakes red, as also seen for solar salterns at the coastline. Since Haloarchaea captured in salt crystals survive for a very long time, the use of natural sea salt was the reason why *Hbt. salinarum* grew on salted fish.

*Hbt. salinarum*,* Haloferax* (*Hfx.*)* mediterranei*,* Halorubrum vacuolatum* and *Haloquadratum* (*Hqr.*)* walsbyi* contain up to 70 spindle- or cylinder-shaped gas vesicles per cell, easily seen as white bodies by phase-contrast light microscopy or transmission electron microscopy (Figure 1b,c). Fourteen *gvp* genes clustered in the genomic vac region are involved in gas vesicle formation in *Hbt. salinarum* and *Hfx. mediterranei* [6], whereas only 12 *gvp* genes (*gvpDE* are lacking*)* are found in the Vac^+^ species *Hrr. vacuolatum* and *Hqr. walsbyi* [7,8,9]. In contrast, *Halopiger xanaduensis*,* Haladaptatus pauchihalophilus* and the haloalkaliphiles, *Natrinema pellirubrum*,* Natrialba magadii* and* Natronobacterium gregoryi*, all contain a vac region lacking *gvpC* and *gvpDE* [10]. These 11 *gvp* genes are sufficient for gas vesicle genesis, but these species are all Vac^−^. It is not known whether their *gvp* gene clusters are generally not expressed or only transcribed under special conditions.

**Figure 2:**
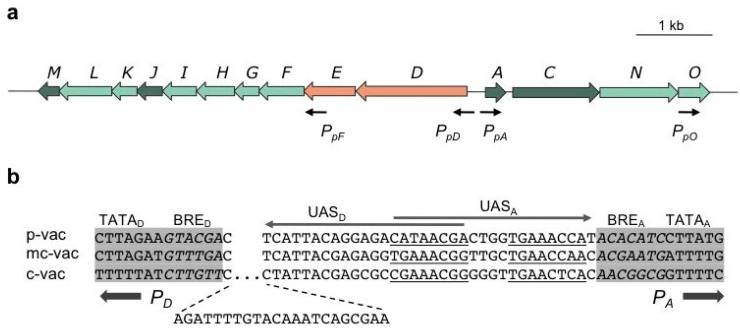
Arrangement of *gvp* genes in p-vac of *Hbt. salinarum* PHH1 (a) and a comparison of the intergenic regions separating *P_D_* and *P_A_* (b). (**a**) The arrows depicting genes are colored as follows: dark green, encoding structural proteins of the A-J-M family and GvpC; light green, encoding accessory Gvp; red, encoding regulator proteins. Black arrows mark the start sites of transcription. (**b**) Comparison of the intergenic regions separating *P_A_* and *P_D_* in p-, mc- and c-vac. A 22-nt insertion occurs in c-vac adjacent to BRE_D_. The 20-nt sequence required for GvpE-mediated activation is underlined (8-nt elements separated by 4 nt of unimportant sequences) in the case of UAS_A _and marked by an arrow. Similar activation elements are found with UAS_D_, pointing in the opposite direction. The TATA-box and BRE sequences (italics) are shaded in grey.

The vac regions have been characterized for the *Hbt. salinarum* strains PHH1, NRC-1, SB3, GN101 and GRB [6,11,12,13]. The latter three strains are more recent natural isolates and genetically stable, whereas PHH1 (DSM 670) and NRC-1 (ATC 700922) are derived from strain collections and exhibit high mutation rates of Vac, Rub and Pum, due to the action of insertion (ISH) elements. The vac regions of PHH1 and NRC-1 were identified using the *gvpA* gene probe of the cyanobacterium, *Calothrix* [11,14]. This gene encodes the major gas vesicle structural protein, GvpA, and is highly conserved between bacterial and archaeal gas vesicle producers. Transformation experiments using the Vac^−^ species, *Hfx. volcanii*, uncovered that *gvpA* is not sufficient for gas vesicle formation and that 12 additional *gvp* genes are required [13,15]. Furthermore, Vac^−^ mutants of PHH1 (and NRC-1) are usually due to the integration of an ISH-element in a 9-kbp region surrounding *gvpA* [13]. The Vac^−^ mutant strain, R1, whose genome sequence has been determined by the Oesterhelt Lab, incurred an ISH3 insertion upstream of p-*gvpA*, preventing the transcription [16]. *Hbt. salinarum* PHH1, R1 and NRC-1 all contain two different *gvp* gene clusters, p-vac (*gvp1* in NRC-1) and c-vac (*gvp2* in NRC-1), whereas SB3, GN101 and GRB contain the c-vac region only [6,12,17]. Expression of p-vac leads to spindle-shaped gas vesicles throughout growth, whereas the cylinder-shaped c-vac gas vesicles occur in the stationary growth phase only. The p-vac region is located on a plasmid and often incurs an ISH element. The 14 *gvp* genes constituting each vac region are arranged in the two oppositely oriented gene clusters, *gvpACNO* and *gvpDEFGHIJKLM* (Figure 2a)*.* This arrangement is typical for p-vac and c-vac, but also for the mc-vac region of *Hfx. mediterranei* [6]. *Hfx. mediterranei* produces cylinder-shaped gas vesicles in the stationary growth phase when grown in 25% salt-media [18]. In contrast, twelve *gvp* genes in *Hrr. vacuolatum* and *Hqr. walsbyi* are arranged as the *gvpACNO-FGHIJKLM* gene cluster with additional DNA inserted between *gvpO* and *gvpF* in *Hqr. walsbyi.*

In this review, I will discuss the expression of the vac regions in *Hbt. salinarum* and *Hfx. mediterranei*, with emphasis on the regulation at the transcriptional and translational level. In addition, experiments to deduce the early steps in the formation of the gas vesicle wall will be presented. Furthermore, I will discuss the applications of gas vesicles in biotechnology and biomedicine.

## 2. Differences Between the *Hbt. salinarum* Strains and the Action of ISH Elements

*Hbt. salinarum* PHH1, R1 and NRC-1 are very closely related, but differ in the arrangement of their plasmids, due to the frequent action of various ISH elements. The nucleotide sequences determined for the chromosomes of NRC-1 and strain R1 differ by twelve point mutations only, but the plasmid sequences are completely rearranged [16]. All three strains contain many ISH-elements, first observed with Vac, Rub or Pum mutants [19,20]. A 1% mutation frequency is found with Vac, but also mutants in Rub or Pum occur at frequencies of 10^−4^ [19]. Rub and Pum are chromosomally encoded, but mutants of these phenotypes always carry insertions in the respective gene regions and also incurred additional alterations in the plasmid DNA, including insertions, duplications, inversions and/or deletions [19,21]. *Hbt. salinarum* NRC-1 contains the *gvp1* gene cluster on the 190-kbp plasmid, pNRC100, whereas the almost identical p-vac region is located on plasmid pHH1 in *Hbt. salinarum* PHH1 or on pHS1 in strain R1 (Table 1) [6,16,22]. The *gvp2* gene cluster resides on the mini-chromosome, pNRC200, in NRC-1, which contains a 145-kbp duplication of pNRC100, including a copy of *gvp1*, whereas c-vac in R1 is located on plasmid pHS3, similar to c-vac in *Hbt. salinarum* PHH1 (Table 1). Gas vesicles due to the expression of c-vac are only found in *Hbt. salinarum* PHH4, which harbors the 35-kbp pHH4, a small derivative of pHH1 lacking p-vac [23,24].

**Table 1:** Characteristics of *Hbt. salinarum* strains.

Strains	Plasmids and vac regions	Vac phenotype	Presence of *tfb* and *tbp*	Reference
PHH1 Vac^+^	pHH1, 150 kbp; p-vac mini-chromosome *; c-vac	spindle-shaped gas vesicles from p-vac in all stages of growth	*tbp*: *A*, *B*, *C*::ISH, *D*, *E*, *F*	[12,19]
			*tfb*: *A*, *B*, *D*, *F*, *G*	[25,26]
PHH4 Vac^+^	pHH4, 35 kbp; mini-chromosome*; c-vac	cylinder-shaped gas vesicles from c-vac in the stationary growth phase	*tbp: E*	[24,27]
			*tfb: A,B,D,F,G*	[25,26]
NRC-1 Vac^+^	pNRC100, 190 kbp; *gvp1* pNRC200, 354 kbp; contains *gvp1* and* gvp2*	spindle-shaped gas vesicles from *gvp1* in all stages of growth	*tbp*: *A*, *B*, *C*, *D*, *E*, *F*	[22]
			*tfb*: *A*,* B*, *C*, *D*, *E*, *F*, *G*; *C + E* on pNRC200	[22]
R1 Vac^−^	pHS1, 147 kbp; p-vac::ISH3 pHS3, 284 kbp; c-vac pHS2, 195 kbp; pHS4, 40 kbp	Vac^−^ phenotype due to an ISH3 insertion upstream of *gvpA* in p-vac	*tbp*: *A*, *B*::ISH, *C*, *D*, *E*, *F*::ISH	[16]
			*tfb*: *A*, *B*, *C*, *D*, *E, F*, *G*, *H*	[16]

* Plasmids or mini-chromosomes in PHH1 were described as the “cccDNA population” [19].

These plasmid alterations also affect the number of genes encoding the general transcription factors, TFB and TBP (Table 1). TFB binds to the BRE sequence adjacent to the TATA-box in the archaeal promoter and recruits, together with the TATA-box binding protein TBP, the RNA polymerase for transcription initiation. Many of the *tfb* and* tbp* genes are plasmid-encoded in *Hbt. salinarum* [16,22]. Six different *tbp* are present, but *tbpB*, *tbpF* or *tbpC in* R1 and PHH1 are inactivated by insertion elements [25] (Table 1). Strain PHH4 even lacks all of the *tbp* genes, except for the chromosomal* tbpE*, which is the only essential *tbp* under standard conditions. Differences are also seen with respect to TFB (Table 1) [26]. All of these variations in *tfb* and *tbp* genes might explain the differences in the amount of gas vesicles in *Hbt. salinarum* PHH1 and NRC-1 when both are grown under non-standard conditions. A systems analysis of the expression of the seven TFBs under different conditions in *Hbt. salinarum* NRC-1 reveals that both the promoter and protein-coding sequences of *tfb* are important in encoding environment-dependent regulatory programs for rapid adaptation to environmental niches [28].

The *Hbt. salinarum* strains, GN101, GRB and SB3, are derived from salt flats in Mexico (GN101), France (GRB) or the USA (SB3) [29]. Their 16S rRNA genes exhibit >99% sequence identity with the 16S rRNA gene of *Hbt. salinarum* PHH1 and NRC-1. All three natural isolates produce cylinder-shaped gas vesicles in late exponential growth due to the expression of c-vac, similar to *Hbt. salinarum* PHH4. The mutation rate with respect to the Vac phenotype is low, since all three contain much less ISH elements compared to PHH1 and NRC-1. The plasmids found here differ from each other, except for the small, multi-copy plasmids, pGN1, pHSB1, and pGRB1, which are homologous (Table 2). All three strains lack plasmids related to the pHH1/pNRC100/pHS1 family and, thus, also p-vac. The lack of the pHH1-type plasmids implies that p-vac might be derived from a lateral gene transfer event.

**Table 2:** Plasmids of *Hbt. salinarum* GRB, SB3 and GN101.

Strains	Plasmids and vac regions	Comments	Reference
GN101 Vac^+^	Plasmids: 39, 43 and 1.7 kbp (pGN1) mini-chromosome; c-vac	Plasmids not related to pHH1/pNRC100/pHS1;1.7-kbp plasmids are homologous	[12,29]
SB3 Vac^±^	Plasmids: 34, 52, and 1.7 kbp (pHSB1) mini-chromosome; c-vac	Plasmids not related to pHH1/pNRC100/pHS1;1.7-kbp plasmids are homologous	[12,29]
GRB Vac^+^	Plasmids: 35, 65, and 1.7 kbp (pGRB1) mini-chromosome; c-vac	Plasmids not related to pHH1/pNRC100/pHS1;1.7-kbp plasmids are homologous	[12,29]

## 3. Regulation at the Level of Transcription Involves GvpE and GvpD

The p-vac region of *Hbt. salinarum* PHH1 contains the 14 *gvp* genes in two oppositely-arranged gene clusters, p-*gvpACNO* and p-*gvpDEFGHIJKLM* (Figure 2a). The intervening sequence between the mRNA start sites of p-*gvpA* and p-*gvpD* is 108 base pairs (bp) in size, with the TATA-BRE elements of the *P_pA_* and *P_pD_* promoters separated by 35 bp (Figure 2b). *P_pA_*, driving the expression of p-*gvpACNO*, is the strongest promoter of p-vac, due to a 70- to 100-fold induction by the endogenous transcriptional activator, GvpE [30]. The co-transcript contains a 20-nt 5'-untranslated region (5'-UTR), and the major 320-nt p-*gvpA* transcript supports the massive GvpA production required to form the gas vesicle wall, which mainly consists of GvpA (>95%). A stem-loop structure occurs between p-*gvpA* and p-*gvpC,* and read-through leads to the minor amounts of the p-*gvpAC*, p-*gvpACN* and p-*gvpACNO* co-transcripts. The stem-loop was thought to be a transcription terminator, but a deletion of this structure results in similarly low amounts of read-through transcripts, as with the wild-type arrangement, implying that the stem-loop stabilizes the p-*gvpA* mRNA, rather than being a termination signal. In case of mc-vac, the half-life of the mc-*gvpA* transcript is almost 90 min, whereas the half-life of the 3-kb mc-*gvpACNO* transcript is 10 min, which is significantly shorter [31]. The p-*gvpC* gene downstream of p-*gvpA* encodes the second structural protein, GvpC, which attaches to the outer surface of the gas vesicles to stabilize the gas vesicle wall. The functions of GvpN and GvpO are not yet known.

The oppositely-oriented* P_pD_* promoter has a weak basal activity that is 20- to 40-fold activated by GvpE. The resulting p-*gvpDE* transcript encodes the two regulatory proteins, GvpD and GvpE. The GvpE protein is one of the few transcriptional activators characterized in Archaea. It dimerizes in solution and resembles a leucine-zipper protein [32]. The GvpE-responsive elements, UAS_A_ and UAS_D_, occur adjacent to the respective BRE and are 20-nucleotides (nt) in size (Figure 2b). Each element consists of two 8-nt sequences separated by 4 nt that are not important for the activation [33,34]. Since *P_pA_* and *P_pD_* initiate transcription in opposite directions, the distal portions of UAS_D_ and UAS_A_ overlap by 7 nt in the center (Figure 2b). *In vitro* studies by protein affinity chromatography yield that GvpE interacts with any TFB and TBP tested [26,35]. Binding constants between GvpE and the various general transcription factors have not been determined. Putative differences in the GvpE-TFB and/or GvpE-TBP affinities might cause alterations in the environment-dependent regulation of *gvp* gene expression. GvpE could stimulate their binding at *P_pA_* and *P_pD _*to enhance the transcription initiation.

**Figure 3:**
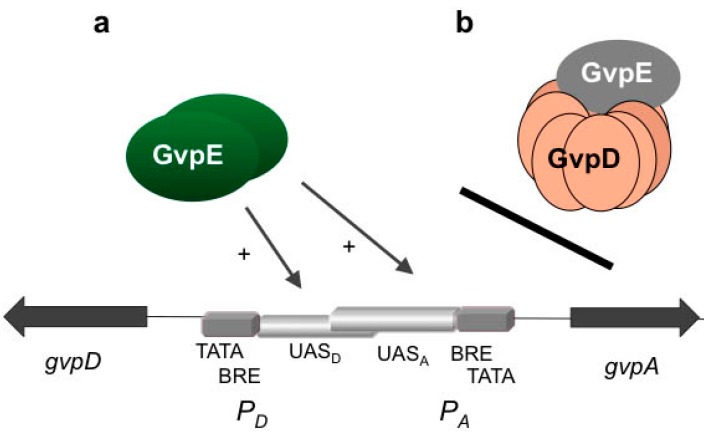
Activation of *P_A_-P_D_* by GvpE (a) and repression by GvpD (b). Schematic representation of the region between *gvpA* and *gvpD* and the two oppositely-oriented promoters *P_A_* and *P_D_*. TATA-box and BRE are shown in grey, and the two UAS elements are partly overlapping in the center in light grey. The reading frames *gvpD* and *gvpA* are represented by dark arrows. (**a**) Activation of transcription by GvpE should involve binding of GvpE, presumably as a dimer, at the respective UAS element and recruitment of TFB, TBP and of the RNA polymerase. (**b**) In the presence of GvpD, the interaction of GvpE-GvpD leads to a strong reduction in the amount of GvpE and the repression of gas vesicle formation.

The presence of GvpE leads to a fast and strong activation of *P_pA_* and *P_pD_* under standard growth conditions, but as soon as GvpD appears, the activation is reduced (Figure 3). GvpD and GvpE are able to interact, and the presence of GvpD leads to an almost undetectable amount of GvpE, as determined by western analysis [36]. The reduction in the amount of GvpE was quantified using the green fluorescent protein smGFP fused to the N-terminus of GvpE [37]. The amount of GFP-GvpE is more than 60% reduced in the presence of GvpD. The fact that a reduction in the amount of GvpE is not observed in the presence of the repression defective GvpD_Mut6_ mutant underlines that a functional GvpD is required for this process [37].

The repressing function of GvpD was initially observed in ∆D transformants carrying the mc-vac region of *Hfx. mediterranei* with a 918-bp deletion in the *gvpD* reading frame (∆D construct) [38]. *Hfx. volcanii* ∆D transformants are filled with numerous gas vesicles, turning the flat and disc-shaped cells into spheres, and the presence of GvpD in ∆D + D transformants reduces the amount of gas vesicles to the wild-type level. GvpD oligomerizes in solution and exhibits similarities to the large family of AAA+ ATPase proteins by possessing a Walker I and II motif to bind ATP and an arginine-rich region [39]. Both motifs are required for the GvpD-induced breakdown of GvpE. The repression defective GvpD_Mut6_ mentioned above carries mutations in the Walker motif, underlining that nucleotide binding is important for the GvpD function [40]. A second arginine-rich region close to the C-terminus of GvpD is also involved in repression, since an alteration of this 494RRR496 sequence to three alanine residues yields the super-repressor, GvpD_3-AAA _[40]. In presence of GvpD_3-AAA_, the fluorescence of GFP-GvpE is reduced to 20% of the original fluorescence in *Hfx. volcanii* transformants [37].

The additional promoters, *P_pF_* and* P_pO_*, of the p-vac region are independent of GvpE and drive the synthesis of accessory Gvp proteins [33]. *P_pO_* yields the essential GvpO throughout growth, but the function of this protein is not yet known. *P_pF_* is active during the exponential and early stationary growth phase and leads to minor amounts of the p-*gvpFGHIJKLM* transcript [41]. The transcript start occurs 177-nt within the *gvpE* reading frame. Since the AUG start codon of *gvpF* occurs 8-nt within *gvpE*, the p-*gvpF-M* transcript contains a 169-nt 5'-UTR. The amounts of the accessory proteins, GvpF through GvpM, are much lower compared to GvpA and GvpC, and except for GvpI and GvpH, all of them are required for the formation of the gas vesicle wall [42].

A very similar GvpE-mediated activation of* P_A_* and *P_D_* occurs in the mc-vac region, whereas in the case of c-vac, *P_cA_* is the only promoter induced by GvpE. *P_cD_* incurred a 22-bp insertion adjacent to BRE, enlarging the distance to UAS_D_ and, thus, abolishing GvpE-activation (Figure 2b) [6,43]. The close distance of UAS-BRE is essential for the activation [34]. The basal activity of *P_cD_* is very weak and yields minor amounts of c-*gvpDEFGHIJKLM* transcripts in late exponential growth. Additional promoters are neither found in mc-vac nor in c-vac.

All of these analyses demonstrate the importance of *P_A_* and *P_D_* for the formation of gas vesicles. The transcriptional activation by GvpE leads to the large amounts of the major gas vesicle structural protein, GvpA. The amount of the activator, GvpE, is reduced in the presence of GvpD and, thus, also, the production of gas vesicles. To investigate the function of the GvpE/GvpD regulatory system in more detail, crystal structures of both proteins are required. Furthermore, additional mutants inactive in regulation will be useful to pinpoint other regions important for the regulatory functions.

## 4. Environmental Factors Influencing Transcription

Oxygen availability, temperature, salt concentration and carbon sources all influence the formation of gas vesicles in Haloarchaea [18,26,44,45,46]. Growth under anoxic conditions is supported by nitrate respiration (*Hfx. mediterranei*) or arginine fermentation in the case of *Hbt. salinarum*. The amounts of the *gvpACNO* and *gvpD-M* transcripts are 10-fold reduced in both species under anoxic conditions, and only a few small gas vesicles are formed [45] (Table 3). *Hfx. volcanii* mc-vac transformants grown by nitrate respiration also show a 10-fold reduced expression. It is interesting to note that aerobic ∆D transformants (*i.e*., mc-vac with a 918-bp deletion in mc-*gvpD*) are filled with numerous gas vesicles, but lack these structures completely when grown under anoxic conditions. Thus, the absence of gas vesicles under anoxic conditions is independent of the repressing protein, GvpD. The extremely large amount of mc-*gvpA* transcripts in ΔD transformants is 10-fold reduced under these conditions, but still large compared to the wild-type. Furthermore, GvpA is present in relatively large amounts in ∆D transformants, implying that the gas vesicle formation is inhibited at the level of assembly [45]. The lower amount of ATP produced under anoxic conditions and/or the smaller amounts of accessory proteins might restrict the formation of gas vesicles under these conditions. However, *Hbt. salinarum* PHH1 and NRC-1 grown by anaerobic respiration with dimethylsulfoxide (DMSO) or trimethylamine-*N*-oxide (TMAO) as the terminal electron acceptor produce many gas vesicles, demonstrating that a lack of oxygen does not always lead to a reduced number of gas vesicles [45,46].

The effect of temperature on gas vesicle formation was studied with *Hbt. salinarum* PHH1 and NRC-1 [26,47]. Heat shock conditions (1 h at 49 °C followed by 3 h at 56 °C and subsequent growth at 37 °C) lead to an eight-fold reduced amount of p-*gvpA* transcripts in PHH1, and the gas vesicle formation stops completely (Table 3). The p-*gvpA* mRNA is rapidly lost during heat shock treatment [26]. A reduction in the *gvpE* mRNA encoding the transcriptional activator, GvpE, might also contribute to the interruption of gas vesicle formation, as determined for NRC-1 [47]. In contrast, PHH4 is able to cope very well with heat shock conditions. A reduction in growth is not observed; nevertheless, the amount of c-*gvpA* drops significantly, and gas vesicle formation stops [26]. It is possible that the secondary structure of the *gvpA* mRNA contributing to the stability of the transcript is destroyed under heat shock conditions. PHH1 and NRC-1 grown at the relatively cold temperature of 15 °C contain very large amounts of gas vesicles. A two-fold increase (PHH1) in the expression of p-*gvpACNO* was determined by quantitative RT-PCR [26], and a 1.8- to 8-fold increase of *gvpACNO* and a three-fold increase in *gvpDE* expression were determined for NRC-1 by microarray analyses [47]. The higher transcription of the *gvp* gene clusters under cold conditions is exceptional, since most housekeeping genes of *Hbt. salinarum* are downregulated at 15 °C. GvpE might activate transcription continuously, since the amount is not reduced. The interaction of GvpD and GvpE (resulting in the degradation of GvpE) might be disturbed or the proteases involved in the breakdown of GvpE are less functional at 15 °C [26].

**Table 3:** Environmental factors influencing gas vesicle formation.

Environmental Factors	Strains and Conditions	Effect on Vac	Effect on *gvp* Transcription
Anaerobiosis	*Hbt. salinarum* p-vac		
	+arginine	Vac^−^	10-fold reduced compared to oxic growth
	+TMAO	Vac^+^	not altered compared to oxic growth
	+DMSO	Vac^+^	not altered compared to oxic growth
Temperature	*Hbt. salinarum* p-vac		
	15 °C	Vac^++^	2-fold increase compared to 37 °C
	heat shock	Vac^−^	8-fold reduced compared to 37 °C
Salt concentration	*Hfx. mediterranei* mc-vac		
	15% salt	Vac^−^	-
	25% salt	Vac^+^	7-fold increase compared to 15% salt
Glucose	*Hfx. mediterranei* mc-vac		
	50 mM	Vac^±^	-
	100 mM	Vac^−^	10-fold reduced compared to standard
	200 mM	Vac^−^	-

The sodium chloride concentration of the medium influences the gas vesicle formation of the moderately halophilic *Hfx. mediterranei.* Gas vesicles are only formed when this species is grown in 17%–30% (w/v) salt media, whereas cells grown in 15% salt media are Vac^−^ (Table 3). The amount of the mc-*gvpA* transcripts is seven-fold enhanced in cells grown in 25%-salt compared to the 15%-salt culture [18]. Glucose also influences the *P_mcA_* promoter activity, since the addition of 50, 100 or 200 mM glucose results in a 10-fold reduction of the basal activity of *P_mcA_*, whereas the activity of the promoter of the housekeeping ferredoxin gene, *P_fdx_*, is almost unaffected [44]. A complete inhibition of gas vesicle formation is observed at glucose concentrations above 100 mM. Maltose and sucrose impose a similar effect, whereas xylose, arabinose, lactose, pyruvate and 2-deoxy-glucose have no influence [44]. Also in this case, ∆D transformants lack gas vesicles in the presence of glucose, demonstrating that the repressing protein, GvpD, is not involved. The function of GvpD as a glucose sensor is thus excluded.

In summary, the GvpE/GvpD regulatory system is often not involved in the transfer of environmental signals to the respective vac region. It appears that both regulatory proteins are mainly required to enhance the gas vesicle formation under standard conditions in *Hbt. salinarum* and *Hfx. mediterranei*. Since most gas vesicle gene clusters of other Haloarchaea lack *gvpDE*, the impact of GvpD and GvpE on *gvp* gene expression is confined to a few species [10]*.*

## 5. Relevance of 5'-UTR and Shine-Dalgarno Sequence for Translation

The transcription of p-vac starts with the p-*gvpFGHIJKLM* in the early exponential growth phase, followed by p-*gvpDE*, p-*gvpACNO* and* p-gvpO*. Except for p-*gvpO*, all transcripts contain a 5'-untranslated region (5'-UTR), with sizes of 20-nt (p-*gvpACNO*), 72-nt (p-*gvpDE*) and 169-nt (p-*gvpFGHIJKLM*). With the exception of p-*gvpA* and p-*gvpO*, all reading frames are preceded by a putative Shine-Dalgarno (SD) sequence (GGAGGUCA) that appears at a distance of 5 to 13 nt of the AUG start codon [48]. The SD element is complementary to a sequence found near the 3' end of the small ribosomal subunit rRNA of *Hbt. salinarum*. In bacteria, SD sequences facilitate the transcript recognition at the 30S ribosomal subunit, whereas in Archaea, their importance is under debate.

An almost perfect SD sequence is found 7 nt upstream of the AUG of the p-*gvpH* reading frame. A 4-nt scanning mutagenesis of this region uncovered that mutations in the SD sequence lead to a 5%–50% reduction in translation, as quantified using *bgaH* encoding a haloarchaeal β-galactosidase as the reporter [48,49]. However, even a complete alteration of this element leads to a residual BgaH activity of 20% compared to the wild-type. Altering the spacing of the SD element of p-*gvpH* with respect to the AUG start codon yields a good translation with distances of 4 to 10 nt, but a spacing of only 1 nt results in the loss of translation. These observations suggest that the SD sequence supports translation, but does not strictly control the expression of a reading frame [48]. These results differ from the analysis of SD sequences in the hyperthermophilic archaeon, *Sulfolobus solfataricus*, where single nucleotide alterations in the SD sequence completely prevent translation* in vitro* [50]. Another investigation of a putative SD sequence in the 5'-UTR of the single *sod* gene of *Hfx. volcanii* shows that partial mutations or even a complete removal of the SD sequence has no effect on the translation efficiency, implying that this sequence has no function [51]. Taking the massive acquisition of bacterial genes in the haloarchaeal founder organism into account [1,2], the SD sequences might be remnants of bacterial origin with little or no influence on the expression of single genes. However, it is remarkable that genes organized in operons, such as in the vac regions, usually contain SD sequences that influence translation, as shown for p-*gvpH* and p-*gvpG* [48].

A much larger influence on translation is even achieved when the 5'-UTR is lacking. Bioinformatic analyses predict that 30% of the haloarchaeal genes are transcribed as leaderless mRNA, including the ferredoxin transcript starting 1 nt upstream of the AUG start codon [52]. Ferredoxin constitutes 2% of the total proteins in *Hbt. salinarum*, and the *fdx* gene is strongly transcribed and efficiently translated. The haloarchaeal expression vector pJAS35 is based on the strong expression of the leaderless transcripts under *P_fdx_* promoter control [53]. In the case of the p-vac region, a deletion of the entire 5'-UTR of p-*gvpH* (*i.e.*, creating a leaderless transcript) results in a 15-fold enhanced translation [48]. Moreover, all leaderless transcripts constructed for p-vac yield a stronger expression compared to their leader-containing counterparts. These results imply that 5'-UTRs regulate gene expression by reducing the translation efficiency. The 5'- or 3'-UTRs of the haloarchaeal transcripts often form secondary structures that stabilize the mRNA (as seen with the p-*gvpA* transcript) or these regions offer binding sites of proteins or small regulatory RNAs, influencing translation initiation. The exploration of the regulatory potential of 5'- and 3'-UTRs is still ongoing and will certainly yield further information. 

## 6. Effect of Deletions and Overexpression of Single *gvp* Genes on Gas Vesicle Formation

The importance of the various Gvp proteins for gas vesicle formation was deduced from *Hfx. volcanii* transformants carrying p-vac constructs that incurred a deletion of a single *gvp* gene (∆X constructs) and also from transformants containing p-vac plus an additional *gvp* gene overexpressed in pJAS35. The deletion analyses determined the eight genes, *gvpA*, *F*, *G*, *H*, *J*, *K*, *L*, *M* and *O*, as essential for the synthesis of gas-filled compartments [42]. Deletion of p-*gvpDE* results in minor amounts of gas vesicles, whereas a deletion of *gvpI*,* gvpH*, *gvpN* or *gvpC* leads to extremely long (∆I), fragile (∆H, ∆C), small (∆N) or large and egg-shaped (∆C) gas vesicles. Complementation of such ∆X transformants by the missing *gvp* gene again results in gas vesicles similar to *Hbt. salinarum* PHH1 [42]. An additional study has been performed with the *gvp1* gene cluster of *Hbt. salinarum* NRC-1 using insertions of a kanamycin-resistance cassette (κ-element) in various *gvp* genes generated in *Escherichia coli* [54]. The effect of this linker-scanning mutagenesis on gas vesicle formation was studied in a pNRC100-deficient *Hbt. salinarum* NRC-1 variant still containing a partially expressed *gvp2* gene cluster. The results of the two studies (deletions* versus* insertions) differ in six out of 14 cases, and possible reasons have been discussed earlier [55]. They include also polar effects of the κ-insertion on the expression of *gvp* genes located downstream of the integration site. 

An overexpression of *gvpF*, *G*, *H*, *I*, *J*, *K*, *L* or *M* in the presence of p-vac does not affect gas vesicle formation, but the presence of large amounts of GvpG, GvpH or GvpM results in Vac^−^ transformants [56]. Since p-vac + GHIJKLM^ex^ transformants (*gvpG-M* inserted in the expression vector pJAS35) produce gas vesicles similar to the wild-type, a balanced amount of the accessory proteins is obviously required for gas vesicle assembly. It is possible that GvpG, GvpH or GvpM interact with other essential Gvp proteins produced in minor amounts by p-vac, thus diminishing the amount(s) of the essential partner protein(s) required for gas vesicle assembly. GvpG and GvpH are soluble, but the hydrophobic GvpM forms aggregate when produced in large amounts, easily seen with GvpM fusions to the green fluorescent smGFP. Such GvpM-GFP (M_GFP_) transformants containing one to two large fluorescent foci per cell. In contrast, cells containing H_GFP_ or L_GFP_ are fully fluorescent [57]. Together with GvpJ and GvpA, GvpM belongs to the A-J-M family of hydrophobic Gvp proteins. P-vac + MX^ex^ transformants (X = G, H, J or L) demonstrate that GvpH, GvpJ or GvpL are able to neutralize the inhibitor effect of GvpM, whereas GvpG is unable to do so. Protein affinity chromatography confirms that GvpM interacts with GvpH, GvpJ or GvpL, but not with GvpG, supporting the hypothesis that M-H, M-J and M-L complexes are formed. L_GFP_ transformants are fully fluorescent, but stable M-L_GFP_ aggregates occur in the presence of GvpM. In contrast, M-H aggregates are neither observed in H_GFP_-M nor in H-M_GFP_ transformants carrying GFP fused to the C-terminus of GvpH (H_GFP_) or GvpM (M_GFP_) [57]. The product could be too small to be visualized as fluorescence foci in the cells. GvpH might prevent the unspecific aggregation of GvpM by keeping this protein “soluble” and, thus, useful for incorporation in the gas vesicle wall. Similar analyses with other accessory Gvp proteins will unravel additional complexes that might occur during gas vesicle assembly.

## 7. Gas Vesicle Wall and Mutations Affecting the Shape

**Figure 4:**
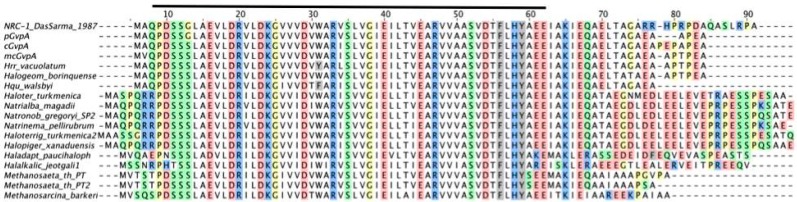
Sequence alignment of GvpA proteins derived from Haloarchaea and methanogens. Identical amino acid residues are highlighted in the following colors: red, negatively charged; blue, positively charged; green, polar, uncharged; yellow, small and variable; grey, aromatic; white, non-polar residues. The bar on top marks the highly conserved central 51 amino acids. The difference in the C-terminus of GvpA (*gvp1*, NRC-1) to the sequence of pGvpA (p-vac, PHH1) is due to a missing G nucleotide close to the 3'-terminus of *gvpA* in* gvp1*. pGvpA and cGvpA are derived from *Hbt. salinarum* PHH1 and mcGvpA from *Hfx. mediterranei*.

**Figure 5:**
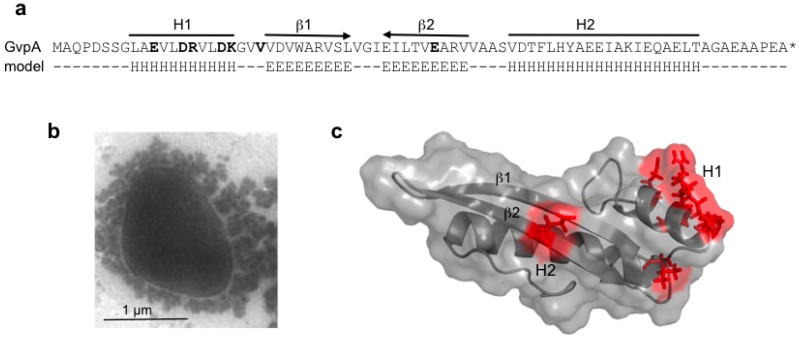
Amino acid sequence of GvpA and the structure derived from the *in silico* modelling. (**a**) Amino acid sequence of pGvpA and the proposed 2D structure (model). H denotes α-helices and E β-strands. The α-helices, H1 and H2, and the anti-parallel β-strands are indicated on top. (**b**) Electron micrograph of a Vac^−^ ∆A + A_mut_ transformant, showing that gas-filled compartments are indeed lacking in such mutants. (**c**) Structure of GvpA, highlighting some positions of ala-substitutions leading to gas vesicle negative ∆A + A_mut_ transformants [63]. The respective single amino acids that are altered are shown in bold in (a).

GvpA constitutes the almost crystalline gas vesicle wall, forming a helix of low pitch seen as ribs running perpendicular to the long axis by transmission electron microscopy [58,59]. The gas vesicle wall is difficult to disaggregate, and the protein constituents are difficult to analyze. Immunological methods and MALDI-TOF mass spectrometry indicate that with the exception of GvpK, all Gvp proteins are present [60,61]. The sequence of the 8-kDa GvpA is highly conserved between archaeal and also bacterial gas vesicle producers (see Figure 4), and differences occur mainly near the N- and C-terminus. GvpA is not post-translationally modified, as demonstrated by protein sequencing and MALDI-TOF mass spectrometry [59,61]. A crystal structure of GvpA is not available due to its hydrophobic nature and high tendency to aggregate. The secondary structure prediction of GvpA suggests a coil-α-β-β-α-coil fold (Figure 5a) [62,63]. Solid-state NMR and Fourier transform infrared spectroscopy (FTIR) with isolated gas vesicles indicates anti-parallel β-sheets, and X-ray analyses and atomic force microscopy imply that the β-strands of GvpA are tilted in the ribs at an angle of 54° [59,62,64]. The C-terminal portion of GvpA is exposed to the outside of the gas vesicles, since a trypsin site and several endopeptidase GluC sites are accessible here, whereas other portions of GvpA are protected [61].

A 3D model was derived from a high-performance *de novo* modelling of mcGvpA [63], and the model was confirmed for the almost identical GvpA of *Hbt. salinarum* NRC-1 [65]. The hydrophobic β-sheet portion most likely faces the gas phase and prevents the precipitation of water molecules entering the gas-filled core. A more hydrophilic portion, including the α-helix H2, constitutes the outer surface facing the cytoplasm. Single amino acid alterations in GvpA, studied in ∆A + A_mut_ transformants for their ability to form gas vesicles, pinpoint essential amino acid positions. Construct ∆A contains all p-vac *gvp* genes, except for *gvpA*, and can be complemented by *gvpA* wild-type or mutant genes [42,63]. Some of the GvpA mutations abolish the formation of gas vesicles,* i.e.*, single alanine substitutions of the polar amino acids in α-helix H1 (Figure 5b,c), whereas other mutations have no effect on gas vesicle formation [63]. Polar residues at the surface might form salt bridges to connect GvpA-GvpA molecules or GvpA with accessory GvpX in the wall. Some GvpA mutants yield extremely long and thin gas vesicles in transformants (GvpA_I34M_ and others) or mini gas vesicles (GvpA_K60A_ and others) that are never or only slowly enlarged [63]. The diversity of gas vesicle structures shows the dependency of the shape on the respective GvpA mutant. Such shape variants are useful to investigate the architecture of this nanocompartment.

## 8. Application of Gas Vesicles

Gas vesicles nanostructures are objects for special applications in biomedical research. The almost crystalline gas vesicle wall provides the structural rigidity to maintain a hollow gas-filled core that is useful as an ultrasonic contrast agent in biomedical research and clinical diagnostics [66]. In contrast to the lipid- or protein-stabilized micro-bubbles, gas vesicles produce a stable ultrasound contrast and provide a much larger mechanical response. The acoustic scattering signal can be also silenced using ultrasound amplitudes exceeding the critical collapse pressure of the gas vesicles. 

Gas vesicles are also applied as reporters for hyperpolarized xenon magnetic resonance imaging (MRI) and enable non-invasive observation of the anatomy and function of organisms [67]. They are capable of chemical exchange saturation transfer interactions. Filled with hyperpolarized ^129^Xe, they allow chemically-amplified gas vesicle detection at picomolar concentrations. Functionalized gas vesicles carrying surface-attached streptavidin antibodies against the HER2 receptor are able to label an HER2-expressing breast cancer cell line, so that these cells are easily distinguishable from others by MRI [67].

In addition, gas vesicles are an effective antigen display system based on a fusion of the peptide of interest to the surface protein, GvpC. The expression of such *gvpC*-fusions in *Hbt. salinarum* yields recombinant gas vesicles displaying these peptides on the surface. The recombinant gas vesicles are easy to isolate by lysis of the haloarchaeal cells and repeated centrifugally accelerated flotation. They are self-adjuvanting, and the antigenic epitopes displayed on the surface stimulate the immune system. Gas vesicles have been used to expose different SIV peptides (Tat, Rev, NefI) [68,69], outer membrane proteins of the pathogen, *Chlamydia trachomatis* [70], or portions of the *Salmonella* SopB antigen [71]. The antiserum raised against the *Chlamydia* proteins is useful to detect the pathogen in sera of *Chlamydia*-positive patients. The *Salmonella* protein, SopB, is injected during the pathogenesis of typhoid, and mice boosted with SopB gas vesicles elicit a protective response to *Salmonella* infection. Such recombinant gas vesicles will be further developed as improved vaccines [71].

## 9. Conclusions

Gas vesicles produced by several microorganisms, including Haloarchaea, have been investigated for a long time. These nanocompartments are easy to isolate, but difficult to disaggregate to investigate the protein constituents of the protein wall. The genes encoding these structures are used as model systems to explore archaeal gene regulation and signal transduction at the level of transcription, translation and protein networks. Current research aims to resolve the structure of the major gas vesicle protein, GvpA, by solid-state NMR, and high resolution cryo-electron tomography on isolated gas vesicles is used to determine the structure of GvpA in the gas vesicle wall. Mutants of GvpA and also of the accessory Gvp support and complement these investigations. Such mutants are also helpful to unravel gas vesicle genesis* in vivo*. More recently, interesting applications of gas vesicles have emerged in biomedical research, taking advantage of the stability of gas vesicles and exploiting them, for instance, as ultrasonic contrast agents.
